# Adherence to CPAP in Randomized Controlled Trials in Obstructive Sleep Apnoea—A Meta-Analysis and Investigation of Predictors

**DOI:** 10.3390/jcm15093264

**Published:** 2026-04-24

**Authors:** Lara Benning, Zoe Bousraou, Matteo Bradicich, Silvia Ulrich, Esther Irene Schwarz

**Affiliations:** 1 Department of Pulmonology, University Hospital Zurich, 8091 Zurich, Switzerland; 2 Faculty of Medicine, University of Zurich, 8006 Zurich, Switzerland

**Keywords:** obstructive sleep apnoea, continuous positive airway pressure, adherence, randomized controlled trials

## Abstract

**Background**: Continuous positive airway pressure (CPAP) is the most effective treatment for obstructive sleep apnoea (OSA). However, CPAP adherence in randomized controlled trials (RCTs) is frequently inadequate, potentially leading to an underestimation of the therapy’s true effect on relevant outcomes. The aim was to identify patient and study characteristics that predict adherence to CPAP therapy in RCTs. **Methods**: PubMed and the existing meta-analyses were searched (1984 to 31 December 2024). A study-level meta-analysis of RCTs comparing CPAP with inactive control in patients with OSA was conducted. Meta-regressions and subgroup analyses (<4 h vs. ≥5 h usage) were undertaken to identify the predictors of CPAP adherence. Risk-of-bias was assessed using the Cochrane RoB-2 tool. **Results**: In 136 RCTs reporting on CPAP use, including 8827 patients with OSA (55 [49.5–59.8] years, 77.4 [61.2–89.2]% male, BMI 31 [28.9–33.2] kg/m^2^, Epworth Sleepiness Scale (ESS) 10.0 ± 2.8, apnoea–hypopnoea-index (AHI) 35.7 ± 13.4/h), mean nocturnal CPAP use was 4.5 ± 1 h. CPAP use of ≥4 h, ≥5 h, and ≥6 h per night was observed in 71.3%, 34.1%, and 7.8% of RCTs, respectively. Higher baseline AHI was the strongest predictor of longer CPAP use in meta-regressions (*p* < 0.001, β = 0.02, 95% CI 0.01–0.04). Baseline AHI was also significantly higher (40.3 ± 12.8 vs. 29.9 ± 12.6) in the ≥5 h vs. <4 h subgroup (*p* < 0.01, large effect size d = 0.84). A higher nightly CPAP usage was more likely in smaller (*p* < 0.05, d = 0.45) and single-centre trials (*p* < 0.05, h = 0.52). Sex distribution, age, BMI, ESS, and follow-up had no significant effect on nightly CPAP use. **Conclusions**: Higher baseline AHI independently predicted longer CPAP use in RCTs, while sleepiness and demographics did not. This study was registered at PROSPERO (CRD420250653394) and received no external funding.

## 1. Introduction

Obstructive sleep apnoea (OSA) is a global health issue affecting nearly 1 billion people [1]. Its overall prevalence, defined by an apnoea–hypopnoea index (AHI) of ≥5 in polysomnography or respiratory polygraphy, ranges from 9% to 38% of the adult population [2]. OSA is more common in men up to the age of 50, and it increases with advancing age, obesity, and in women after menopause [2,3]. It is a heterogeneous disorder with a variety of underlying mechanisms, along with diverse clinical and poly(somno)graphic features, contributing to different endotypes and phenotypes (e.g., OSA severity, positional OSA, sleepy vs. non-sleepy OSA) [4]. Diverse OSA types respond differently to different treatment approaches like continuous positive airway pressure (CPAP), mandibular advancement device (MAD), or positional therapy. Since its introduction in 1981 [5], CPAP is the most potent and state-of-the-art treatment [6], which, if effectively used, improves sleepiness, quality of life, blood pressure control, and long-term vascular outcomes [7,8,9,10,11]. The American Academy of Sleep Medicine (AASM) guidelines strongly recommend using CPAP or autoCPAP (APAP) for long-term treatment of OSA in adults [12]. OSA is often more severe during rapid eye movement sleep, which occurs predominantly in the second half of the night [13]. This highlights the importance of CPAP usage throughout the entire night, although this is often not achieved due to intolerance (e.g., side effects) or lack of acceptance [14,15].

Insufficient CPAP adherence (both in terms of frequency and duration of nightly use) is not only a concern in real-world settings, but it is also common in randomized controlled trials (RCTs). In RCTs examining the effects of CPAP on outcomes in OSA, the treatment effect may be underestimated or diluted due to inadequate CPAP utilization, leading to erroneous conclusions about the significance of untreated OSA or the effectiveness of OSA treatment. Meta-analyses and on-treatment analyses have been employed to more accurately estimate the treatment effect of CPAP and address the issue of insufficient therapy adherence. Several meta-analyses have identified a significant treatment effect on outcomes only in the subgroup of OSA patients using CPAP for at least 4 h per night [11,16]. An individual patient data meta-analysis on the effect of CPAP on recurrent major adverse cardiac or cerebral events (MACCEs) showed no risk reduction in the CPAP group compared to the control group [11]. However, an on-treatment analysis revealed a lower risk of MACCEs in patients using CPAP for at least 4 h per night, with a significant reduction in cardiovascular mortality and stroke [11]. The importance of CPAP adherence in achieving a significant treatment effect has been demonstrated for a wide range of outcomes, including blood pressure [17,18], quality of life [19], daytime sleepiness [20], and metabolic [21] changes. The commonly cited threshold of a minimum of 4 h of nightly CPAP use is considered to be an acceptable level of adherence, and it is associated with a positive impact on outcomes. However, several studies suggest that using CPAP for longer than 4 h per night may have a greater effect, depending on the outcome [18,22,23,24]. However, it is also evident that this effect approaches a plateau with additional hours of CPAP use beyond a certain threshold no longer results in a proportional improvement [10]. RCTs are the most rigorous studies for investigating the effects of interventions such as CPAP. However, they are limited by reduced generalizability due to highly selected patient populations, and the treatment effect is often underestimated because of low CPAP adherence, which is even lower than in real-life OSA cohorts. Understanding which patient, sleep study, and trial characteristics are associated with longer nightly CPAP use is crucial for the design of future interventional trials. In this initial approach, this study-level meta-analysis aims to identify patient (age, sex distribution, body mass index [BMI], and Epworth Sleepiness Scale [ESS] at baseline or its change), sleep study (AHI), and study (design, follow-up duration, sample size, and outcome) characteristics that independently predict CPAP adherence in RCTs of OSA.

## 2. Materials and Methods

### 2.1. Study Registration, Eligibility Criteria, and Data Extraction

This study-level meta-analysis was performed according to the preferred reporting items for systematic reviews and meta-analyses (PRISMA) [25]. It was previously registered in PROSPERO (CRD420250653394). A systematic search on the PubMed (MEDLINE) database (1984 to 31 December 2024) was conducted and references of existing meta-analyses on CPAP were screened. Details on the literature research can be found in the Supplementary Materials (Supplementum S1.1). After the removal of duplicates, the studies were checked for inclusion criteria using titles and abstracts. The full texts of studies that were considered suitable were reviewed by two authors each. The following inclusion criteria were applied: RCTs (parallel or crossover), adult patients ≥ 18 years, OSA (AHI ≥ 5 per hour in polysomnography or respiratory polygraphy), intervention group received CPAP, control group received either no treatment, sham-CPAP (CPAP at non-therapeutic pressure level), conservative treatment (i.e., lifestyle education) or oral placebo, objective CPAP usage time was reported, follow-up of at least 4 weeks, and article in English. If the data were published more than once, the earliest and most comprehensive dataset was used. If a protocol referred to a previous publication in terms of study design or patients’ characteristics, these data were used.

The data were extracted using a standardized spreadsheet recording study details, patient demographics, ESS, sleep study data, and CPAP use (hours/night, nights/week, %patients ≥ 4 h, pressure, and device AHI). If multiple follow-up periods were reported, the longest (up to 52 weeks) was used. Missing data were marked as unavailable. Two authors estimated the risk-of-bias for each publication using the Cochrane Collaboration’s risk-of-bias tool. For crossover trials, an appropriate tool was employed [26]. Concerns arose when there was a pre-randomization run-in, high drop-out, non-compliance, compliance encouragement via phone, subjective compliance reporting, or lack of wash-out in crossover trials. Disagreements were resolved by consensus.

### 2.2. Outcomes

The primary outcomes of interest were patient (age, sex, BMI, and ESS (change)), sleep study (AHI), and study (design, follow-up time, sample size, and outcome) characteristics, which independently predicted mean CPAP use (h/night). Secondary outcomes were patient (age, sex, BMI, and ESS (change)), sleep study (AHI), and study (design, follow-up time, sample size, and outcome) characteristics predictive of >50% of patients using CPAP at least 4 h per night (>50% patients ≥ 4 h/night). Potential predictors were predefined based on clinical relevance and consistent and frequent reporting in RCTs.

### 2.3. Statistical Analysis

A study-level meta-analysis was performed with effect sizes weighted by each study’s standard error. The mean change from baseline to follow-up was used as reported or calculated as the arithmetic difference between baseline and follow-up. The data were tested for normal distribution suing Shapiro–Wilk test and quartile–quantile plot. Meta-regressions (mixed effects models) were run using restricted maximum likelihood (REML) and Hartung–Knapp modification [27]. In additional multivariable meta-regression models, adjustments for clinically meaningful moderator variables (after checking for multicollinearity) were tested through multi-model inference. The best-fitting model was chosen based on model evaluation metrics, including Akaike’s information criterion (AIC), R^2^, and analysis of variance (ANOVA). Heterogeneity was tested using the I^2^ Index. Subgroup analyses were performed for CPAP use cut-off values of (e.g., <4 h vs. ≥5 h). The *t*-test for independent samples, alternatively the Welch test for unequal variances (for the comparison of two groups) or the one-way ANOVA with subsequent Bonferroni correction (for the comparison of >2 groups), were used to compare subgroups for continuous parametric variables. The Mann–Whitney-U test (for the comparison of two groups) or the Kruskal–Wallis test with subsequent Bonferroni correction (for the comparison of >2 groups) was used for the comparison of subgroups for continuous non-parametric variables. The Chi^2^ test with subsequent Bonferroni correction was used to compare subgroups for nominal variables. Clinically meaningful cut-offs were determined using receiver operating characteristic (ROC) curves, Youden index, and the area under the curve (AUC). Sensitivity analyses excluded crossover studies or those with risk-of-bias concerns. Publication bias was assessed using a funnel plot and Egger’s regression test. Statistical significance was set at α < 0.05, with effects reported at 95% CI. SPSS (version 31.0.2.0 (126), IBM Corp., Armonk, NY, USA) and RStudio (version 2025.09.2 (418), PBC, Boston, MA, USA) were used for all analyses.

## 3. Results

### 3.1. Included Studies and Patients

**Figure 1:**
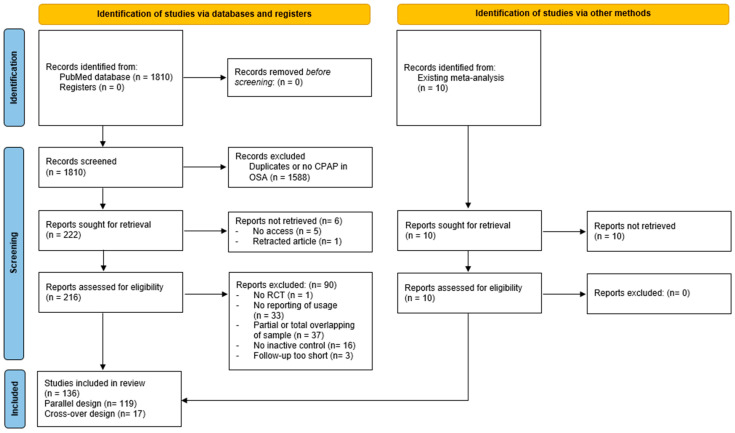
Study flowchart. A total of 136 RCTs were included. RCT = randomized controlled trial.

A total of 1820 records were screened for eligibility, of which 232 were deemed suitable for full-text review. Ultimately, 136 RCTs, involving 8805 patients on CPAP, were included in the final analysis (Figure 1). Of these, 119 RCTs employed a parallel design (8280 patients on CPAP), while 17 RCTs used a crossover design (525 patients on CPAP).

A total of 67.2% of the included studies were single-centre. The median follow-up duration was 13 (8–26) weeks. The primary outcomes of the included RCTs were cardiovascular (46%), OSA symptoms (16.1%), quality of life (8.8%), metabolic (15.3%), and neurocognitive/motoric (13.9%) outcomes. A total of 77.4 (61.2–89.9) % of the study population was male. The patients had a median age of 54.9 (49.5–59.8), a median BMI of 31 (28.9–33.2) kg/m^2^, a mean AHI of 35.7 ± 13.4/h, and a mean ESS of 10.0 ± 2.8. CPAP was used for ≥4 h, ≥5 h, and ≥6 h per night in 71.3%, 34.1%, and 7.8% of the included RCTs, respectively. Mean CPAP usage was 4.5 ± 1 h per night, with 61.9 ± 19.7% of patients using CPAP for at least 4 h/night. Hypertension and diabetes were the most common comorbidities. The effect of CPAP on ESS was −3 (−4.4–−2.4) points. Further characteristics are shown in Table 1.

### 3.2. Predictors of CPAP Usage Hours

**Figure 2:**
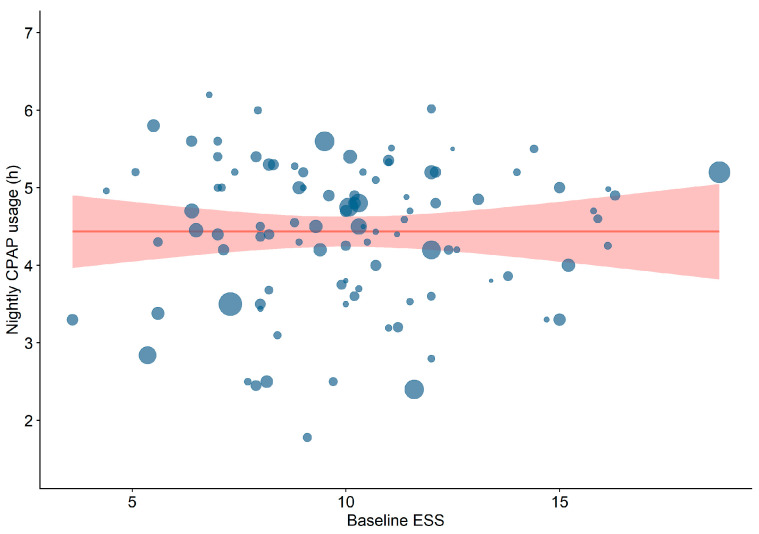
Meta-regression on the effect of baseline ESS on nightly CPAP usage hours. Red area represents 95% confidence interval. Bubble sizes are inversely proportional to their study’s standard error. CPAP = continuous positive airway pressure. ESS = Epworth Sleepiness Scale.

**Figure 3:**
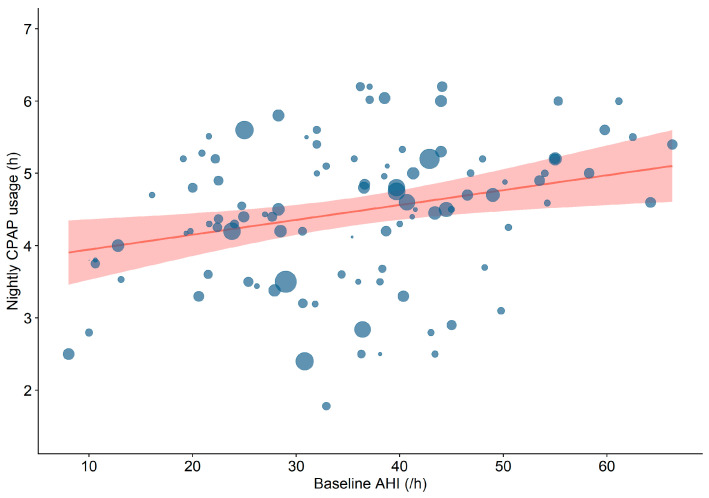
Meta-regression on the effect of baseline AHI (/h) on nightly CPAP usage hours. Red area represents 95% confidence interval. Bubble sizes are inversely proportional to their study’s standard error. AHI = apnoea–hypopnoea-index. CPAP = continuous positive airway pressure.

Among patient (sex distribution, age, BMI, ESS (Figure 2), ESS change, and AHI) and study (mono- vs. multicentre, follow-up time, and sample size) characteristics tested in meta-regressions, a higher baseline AHI was the strongest predictor of longer nightly CPAP usage (*p* < 0.001, β1 = 0.02, 95% CI 0.01–0.04, R^2^ = 11.3, AIC = 272, Figure 3). When accounting for various clinical moderator variables (sex distribution, age, BMI, and type of sleep study), the model for AHI improved after adjusting for type of sleep study and BMI (*p* < 0.01, β1 = 0.02, 95% CI 0.008–0.04, R^2^ = 12.8, AIC = 254). Furthermore, after controlling for ESS, the model remained significant and even improved (*p* < 0.01, β1 = 0.02, 95% CI 0.007–0.04, R^2^ = 8.9, AIC = 229). Single-centre studies (*p* < 0.05, β1 = 0.37, 95% CI 0.004–0.73) and smaller sample size (*p* < 0.05, β1 = −0.001, 95% CI −0.002–−0.0001) were also statistically significant predictors of longer nocturnal CPAP use (Table 2). We performed a sensitivity analysis excluding an outlying study regaring the sample size (McEvoy et al.) and the results stayed robust.

**Figure 4:**
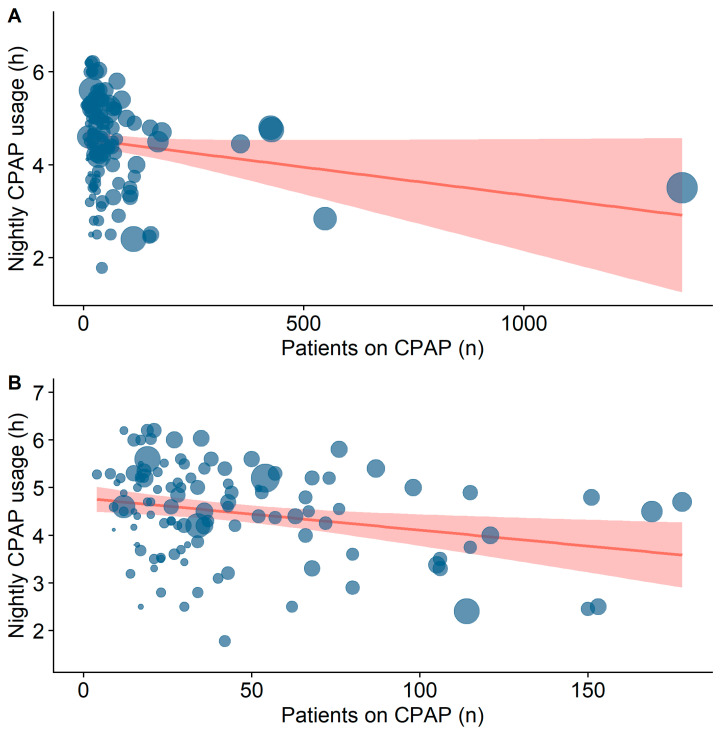
(**A**) Meta-regression on the effect of number of patients on CPAP on nightly CPAP usage hours. Red area represents 95% confidence interval. Bubble sizes are inversely proportional to their study’s standard error. (**B**) *X*-axis is limited for better visualization. CPAP = continuous positive airway pressure.

In subgroup analyses (≥5 h vs. <4 h CPAP use), in which potential predictors of CPAP use (sex distribution, age, BMI, ESS, ESS change, AHI, mono- vs. multicentre, follow-up time, sample size, and outcome group) were tested, baseline AHI was significantly higher in the ≥5 h subgroup compared to the <4 h subgroup (40.3 ± 12.8/h vs. 29.9 ± 12.6/h; *p* = 0.002) with a large effect size (d = 0.84). The ROC analysis identified a baseline AHI of 38.4/h (AUC 0.7, 95% CI 0.57–0.82) as the cut-off between ≥5 h vs. <4 h CPAP use. Additionally, a smaller sample size (23 (17–36.5) vs. 34 (23–105), *p* = 0.01, d = 0.45, Figure 4) and a higher proportion of single-centre studies (81.8% vs. 56.8%, *p* = 0.02, h = 0.52) were observed in the ≥5 h subgroup compared to the <4 h subgroup.

**Figure 5:**
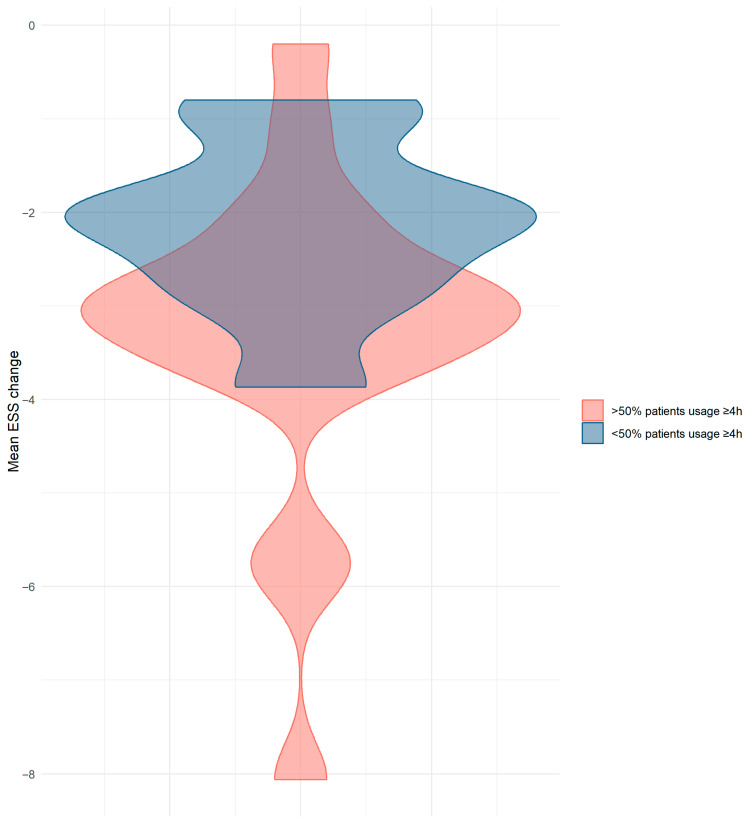
Violin plot of mean ESS change in different CPAP usage groups. CPAP = continuous positive airway pressure. ESS = Epworth Sleepiness Scale.

When comparing the groups with ≥50% vs. <50% patients using CPAP for ≥4 h nightly, baseline AHI was significantly higher (39.3 ± 14.1/h vs. 29.8 ± 10.4/h, *p* = 0.02, d = 0.73) in studies with ≥50% patients achieving ≥4 h usage. Additionally, the reduction in ESS was significantly greater (−3 (−3.6–−2.6) vs. −2 (−2.7–−1.9), *p* = 0.04, d = 0.73) in studies with ≥50% patients using CPAP for ≥4 h nightly (Figure 5).

### 3.3. Risk-of-Bias and Sensitivity Analysis

Of 119 parallel studies, 82 were rated as “low concern”, 33 as “some concern”, and four as “high risk”. Of 17 crossover studies, eight, seven, and two were classified as “low concern”, “some concern”, and “high risk”, respectively (Tables S1 and S2). In sensitivity analyses, studies rated as “some concern” and “high risk” were excluded. All analyses remained robust, except for the comparison of single-centre vs. multicentric studies, which was no longer significant. The sensitivity analysis for all crossover studies did not result in any changes.

### 3.4. Reporting and Publication Bias

Based on the funnel plot and Egger regression test, there was no evidence of publication bias (Figure S1). The included RCTs did not always provide sufficient information on patient or OSA characteristics, or on CPAP use and specifications (especially %nights/week, %patients ≥ 4 h/night, and %nights ≥ 4 h/night) (Table 3). A total of 33 (19.5%) otherwise eligible RCTs did not report any data on nocturnal CPAP use (Figure 1).

## 4. Discussion

In this study, we aimed to identify the predictors of CPAP adherence in RCTs of OSA patients. The results of our meta-analysis, which included 136 RCTs with 8827 patients, highlight that baseline AHI is the strongest predictor of CPAP adherence. Specifically, a higher baseline AHI was associated with longer nightly CPAP use, with a significant difference observed between subgroups using CPAP for ≥5 h versus <4 h per night. Interestingly, factors such as sex distribution, age, BMI, ESS scores, and follow-up duration did not significantly influence CPAP use. Additionally, smaller and single-centre trials showed higher CPAP usage, suggesting that study design may also play a role in adherence. These findings underscore the importance of considering the baseline AHI when designing future RCTs and highlight the need for strategies to improve adherence, particularly in patients with lower AHI.

As this meta-analysis demonstrates that the most significant independent predictor of prolonged CPAP use in RCTs of OSA is a higher baseline AHI, it could be concluded that future RCTs investigating the treatment effects of CPAP on relevant outcomes in OSA may prioritize patients with severe OSA, as defined by the AHI. Similarly, in other study designs, such as in observational studies, severe OSA (AHI ≥ 30/h) was associated with higher CPAP adherence compared to moderate and mild OSA [28]. This meta-analysis revealed a baseline AHI cut-off value of 38/h for CPAP adherence of ≥ 5 h per night in RCTs. However, the conclusions of this meta-analysis are primarily applicable to the study population included, which was predominantly composed of middle-aged, obese males, and may not be fully generalizable to other demographic groups, particularly women. Unfortunately, other relevant parameters of OSA severity, particularly hypoxaemia metrics, such as t < 90 or hypoxemic burden [29], were reported in only a limited number of studies, preventing an investigation of their influence on CPAP use. In addition to OSA severity parameters, a recent review of CPAP adherence predictors, encompassing both RCTs and non-randomized studies, emphasized that there is no single predictor but rather multiple contributing factors. The best predictors included, among others, residual AHI and CPAP use during the first two weeks of the treatment, while evidence regarding AHI, ESS, BMI, and age remained conflicting. Interface, side effects, comorbidities, and sex were not found to be predictive [30]. ESS, alongside the AHI, has been found to be independently related to CPAP adherence and long-term use in both retrospective and prospective studies [28,31]. However, contrary to the expectations, daytime sleepiness as measured by ESS did not emerge as an independent predictor of CPAP adherence in our meta-regression. One potential explanation for this could be the nature of our meta-analysis, which is based on study-level aggregate data rather than individual patient data. Nevertheless, incorporating ESS into the AHI model did improve the overall model fitting. It is worth noting that patient perception of improvement in daytime sleepiness appears to be a strong motivator for continued CPAP use [32]. In our analysis, the ESS change was significantly more pronounced in RCTs where over 50% of patients used CPAP for ≥4 h per night.

However, while this 4 h threshold is frequently reported, evidence suggests that a higher duration of CPAP use may be required to observe significant benefits across a range of outcomes [10,18,33]. This suggests that while >4 h of CPAP use remains a commonly accepted benchmark, future studies and clinical guidelines may need to reconsider and potentially revise this threshold to reflect the duration required for optimal therapeutic efficacy.

In this meta-analysis of CPAP RCTs, the overall CPAP usage during a median follow-up of 13 weeks (~90 days) was 4.5 h per night. Real-life data from a large cloud database with over 2.6 million patients had a 90-day CPAP adherence of 5.1 h/night (median 93% of nights used), indicating a lower adherence in RCTs than in real-life [34]. However, not only usage hours but discontinuation over time plays a role in this. Tracking withdrawal rates in CPAP RCTs is challenging due to inconsistent reporting and short follow-up time. Non-CPAP treatments for OSA, such as MADs or positional therapy, may be alternatives, but they are often less effective. A meta-analysis of MAD use showed an adherence of 6.2 h per night after 5 months, with custom-made MADs being used more frequently [35]. A key advantage of CPAP RCTs is the objective measurement of device usage, which allows for the analysis of treatment effects across different usage groups. This is not available with therapies like MADs, where patients tend to overestimate usage by 1.2 h per night [36]. Compliance with positional therapy also decreases over time, with only 42% adherence after 6 months and less than 10% after 2.5 years [37,38]. Adherence to other treatments, such as treatments for hypertension, is similarly problematic in both RCTs and real-life settings [39].

As patients with OSA are a heterogeneous group, it is essential for future RCTs on OSA therapies to include phenotypes of OSA that are appropriate for the specific intervention and outcome being investigated. From this analysis, it can be concluded that patients with severe OSA are more likely to be selected for CPAP RCTs, as without this, lower CPAP utilization and an underestimation of the treatment effect may be anticipated. Additionally, an important factor to consider may be early adherence, as this is a strong predictor of long-term use, explaining up to 50–86% of the variance [40,41]. Within just a few days, intermittent users tend to distinguish themselves from consistent users [42,43]. Motivational interventions have been shown to be more effective than standard care or educational programmes, particularly in the short term [44], and telemonitoring with subsequent home visits or mask adjustments for those with poor early adherence can have a positive impact [45]. Early follow-up, encouragement, and troubleshooting may help improve CPAP adherence in RCTs beyond the baseline inclusion criteria.

A major limitation of this meta-analysis is its reliance on study-level aggregate data, which provides less precise predictor analysis compared to a meta-regression using individual patient data. Future research should focus on patient-level data, especially to assess gender differences. However, the large number of included studies and the broad range of key predictors analyzed help mitigate this limitation. A further limitation is that only variables that were reported in most RCTs could be analyzed as factors influencing CPAP use. Some interesting markers, e.g., time with SpO_2_ < 90% or hypoxemic burden, were not reported in most CPAP RCTs. Due to insufficient reporting in the RCTs, potentially important predictors of CPAP use, such as CPAP modality (autoCPAP vs. constantCPAP), CPAP, type of interface (nasal vs. oro-nasal mask) and leakage, could not be investigated. Such interface and device factors may influence CPAP acceptance and tolerance [46]. Due to variations in study design, a heterogeneous population, and inconsistent reporting, heterogeneity remained high even after adjusting for confounders. Length of follow-up time varied across studies, leading to differences in predictors at different follow-up times. Due to high heterogeneity, we did not perform analyses on this aspect. The results comparing single-centre and multicentric studies should be interpreted with caution, as they were not robust in sensitivity analyses.

## 5. Conclusions

Using study-level aggregate data, this analysis found that a higher baseline AHI is the strongest predictor of longer CPAP usage in RCTs, while factors such as sleepiness and patient characteristics did not have a significant effect. Measures must be implemented in RCTs to improve CPAP adherence, particularly among patients with mild to moderate OSA, in order to ensure accurate evaluation of treatment efficacy. Studies that successfully reduced daytime sleepiness, as measured by changes in the ESS, were associated with a higher proportion of patients using CPAP for at least 4 h per night. These findings could help optimize future trial designs.

## Figures and Tables

**Table 1:** Patient anthropometric, sleep study, and study characteristics.

Variable	Mean (± SD)/Median (IQR)/n (%)
**Patient Anthropometrics**	
Sex (%men)	*n* = 134, 77.4 (61.2–89.9)
Age (years)	*n* = 134, 55 (49.5–59.8)
BMI (kg/m^2^)	*n* = 128, 31 (28.9–33.2)
Neck circumference (cm)	*n* = 55, 41.8 ± 2.6
Comorbidity (%)	
- Arterial hypertension	*n* = 76, 62.2 ± 30.7
- Diabetes mellitus type 2	*n* = 73, 31.6 ± 34
- Heart failure	*n* = 25, 0 (0–41.4)
- Coronary artery disease	*n* = 36, 9.7 (0–45.1)
- Stroke	*n* = 28, 6.4 (2.3–31)
Smoking status	
- Active	*n* = 60, 20.8 (7.8–37.2)
- Previously	*n* = 21, 37.3 ± 20.7
- Never	*n* = 23, 38 (32.9–64.1)
**OSA and sleep study characteristics**	
Type of sleep study (%)	*n* = 128
- Polysomnography	75.8
- Respiratory polygraphy	24.2
ESS	*n* = 113, 10 ± 2.8
AHI (/h)	*n* = 118, 35.7 ± 13.4
ODI (/h)	*n* = 60, 28.7 ± 13.1
Mean nocturnal SpO_2_ (%)	*n* = 28, 92.7 (91.1–94)
Nadir nocturnal SpO_2_ (%)	*n* = 58, 79 (75.3–82.1)
Time below 90% (%)	*n* = 51, 16.3 ± 19.2
ESS change	*n* = 65, −3 (−4.4–−2.4)
AHI change (/h)	*n* = 42, −28.8 ± 13.8
**Study Characteristics**	
Sample size CPAP group (*n*)	*n* = 136, 30 (18–62)
Follow-up time (weeks)	*n* = 136, 13 (8–26)
Centre	*n* = 136
- Single-centre	67.2
- Multicentre	32.9
Type of control	*n* = 136
- No CPAP/standard care	57.4
- Sham-CPAP	38.2
- Placebo oral appliance	0.74
- Placebo oral tablet	3.68
Main Outcome	*n* = 136
- Cardiovascular	46
- OSA symptoms	16.1
- Metabolic	15.3
- Neurocognitive/motoric	13.9
- Quality of life	8.8
**CPAP device data**	
Usage h/night	*n* = 128, 4.5 ± 1
Usage ≥ 4 h/night (% patients)	*n* = 59, 61.9 ± 19.7
Usage ≥ 4 h/night (% nights)	*n* = 12, 64 (57.3–69.7)
Pressure (cmH_2_O)	*n* = 65, 9.5 ± 1.7
Device AHI (/h)	*n* = 21, 4 ± 2.1

AHI = apnoea–hypopnoea index. CPAP = continuous positive airway pressure. BMI = body mass index. ESS = Epworth Sleepiness Scale. ODI = oxygen desaturation index. OSA = obstructive sleep apnoea.

**Table 2:** Meta-regression analyses.

Variable	β_1_	SE	95% CI	p-Value	df	R^2^ (%)	I^2^ (%)
**Patient Characteristics**							
Sex (male)	0.002	0.004	−0.007–0.01	0.69	116	0	94.4
Age	0.02	0.01	−0.009–0.04	0.2	116	0.5	94.3
BMI	0.03	0.03	−0.02–0.09	0.22	111	0.6	94.3
ESS	0.01	0.03	−0.05–0.07	0.7	99	0	93.9
ESS change	−0.02	0.04	−0.11–0.07	0.7	56	0	91.4
AHI	0.02	0.007	0.01–0.04	**0.001**	101	11.4	94.3
AHI (controlled for sleep study + BMI)	0.02	0.007	0.01–0.04	**0.002**	91	12.8	93.8
AHI (controlled for ESS)	0.02	0.007	0.007–0.04	**0.005**	84	8.9	93.7
**Study Characteristics**							
Sample size CPAP	−0.001	0.001	−0.002–−0.0001	**0.04**	118	3.4	93.8
Follow-up time (weeks)	−0.003	0.002	−0.007–0.001	0.12	142	1.4	95.6
Single-centre	0.37	0.18	0.004–0.73	**0.005**	118	3.4	94.1

AHI = apnoea–hypopnoea index. CI = confidence interval. CPAP = continuous positive airway pressure. BMI = body mass index. df = degrees of freedom. ESS = Epworth Sleepiness Scale. SE = standard error. Bold formatting indicates significance.

**Table 3:** Number (%) of studies reporting on specific characteristics.

Variable	Number (%) of Studies Reporting
**Patient Characteristics**	
Sex	134 (97.8)
Age	134 (97.8)
BMI	128 (93.4)
ESS	113 (82.5)
ESS change with CPAP	65 (47.5)
AHI	118 (86.1)
AHI change with CPAP	42 (30.7)
**CPAP Usage and Specifications**	
Usage (h/night)	128 (93.4)
Usage (%nights/week)	14 (10.2)
%patients ≥ 4 h/night	59 (43.1)
%nights ≥ 4 h/night	12 (8.8)
Pressure (cmH_2_O)	65 (47.5)
Device AHI (/h)	21 (15.3)

AHI = apnoea–hypopnoea index. CPAP = continuous positive airway pressure. BMI = body mass index. ESS = Epworth Sleepiness Scale.

## Data Availability

The data are available from the corresponding author upon reasonable request.

## Supplementary Materials

The following supporting information can be downloaded at: https://www.mdpi.com/article/10.3390/jcm15093264/s1, PRISMA 2020 Checklist; PRISMA 2020 for Abstracts Checklist [47]; Supplementum S1: Details on the literature research.

## Abbreviations

The following abbreviations are used in this manuscript:

AASM	American Academy of Sleep Medicine
AIC	Akaike’s information criterion
AHI	apnoea–hypopnoea-index
ANOVA	analysis of variance
APAP	auto positive airway pressure
AUC	area under the curve
BMI	body mass index
CPAP	continuous positive airway pressure
ESS	Epworth Sleepiness Scale
MACCE	major adverse cardiac or cerebral event
MAD	mandibular advancement device
ODI	oxygen desaturation index
OSA	obstructive sleep apnoea
PRISMA	preferred reporting items for systematic reviews and meta-analyses
RCT	randomized controlled trial
REM	rapid eye movement sleep
REML	restricted maximum likelihood
ROC	receiver operating curve
