# Supplementary material for: Adherence to CPAP in Randomized Controlled Trials in Obstructive Sleep Apnoea—A Meta-Analysis and Investigation of Predictors
Source: J Clin Med. 2026 Apr 24;15(9):3264. doi: 10.3390/jcm15093264 (PMC13164055; doi:10.3390/jcm15093264)
Supplement: Supplementary file 1 [file jcm-15-03264-s001.zip › Supplementum S1_LB.pdf]

Journal of
Clinical Medicine

Systematic Review

Adherence to CPAP in Randomized Controlled Trials in Obstructive Sleep Apnoea—A Meta-Analysis and Investigation of Predictors

Lara Benning [1,2], Zoe Bousraou [1,2], Matteo Bradicich [1], Silvia Ulrich [1,2] and Esther Irene Schwarz [1,2,*]

[1] Department of Pulmonology, University Hospital Zurich, 8091 Zurich, Switzerland
[2] Faculty of Medicine, University of Zurich, 8006 Zurich, Switzerland
* Correspondence: estherirene.schwarz@usz.ch

Supplementum S1

Supplementum S1.1: Systematic literature database search

PubMed search: (obstructive sleep apn*[Title/Abstract] OR OSA[Title/Abstract] OR OSAS[Title/Abstract] OR SAS[Title/Abstract] OR sleep-dis*[Title/Abstract]) AND (CPAP[Title/Abstract] OR continuous positive[Title/Abstract] OR PAP[Title/Abstract] OR APAP[Title/Abstract] OR positive airway pressure[Title/Abstract]) AND rando* => 1810 references.

Existing meta-analyses: records from references from existing meta-analyses on CPAP in OSA => 10 additional references.

The period for literature research was from 1984 to 31 December, 2024. In total, 136 articles were included in the final analysis [1-136].

Supplementum S1.2: Overview on risk of bias

Table S1.

Risk of bias	n RCTs (parallel/crossover)
Low concern	82/8
Some concern	33/7
High concern	4/2

Supplementum S1.3: Study characteristics

Table S2. Characteristics of included RCTs.

Author, Year	Risk of bias	Study design	Centre	Follow-up (weeks)	Primary outcome	Sample size analysed (CPAP)	Gender (male %)	Age (mean)	BMI (mean kg/m^2)	AHI (mean/h)	ESS (mean)	CPAP usage (mean h/night)	CPAP usage (%patients≥4h/night
Aaronson, 2016	Some	Parallel	Mono-	4	Neurocognitive	17	60	61.1	28.1	38.1	NA	2.5	35
Alessi, 2021	Low	Parallel	Mono-	26	OSA symptoms	62	98.4	62.8	NA	36.3	9.7	2.5	NA
Arias, 2005	Some	Cross-over	Mono-	12	Cardiovascular	27	100	52	30.5	44	NA	6	NA
Arias, 2006	Some	Cross-over	Mono-	12	Cardiovascular	21	96	51	30.9	44.1	NA	6.2	NA
Aurora, 2023	Some	Parallel	Mono-	13	Metabolic	87	57	58.4	33.8	NA	10.1	5.4	77
Baillieul, 2021	Low	Parallel	Mono-	8	Neurocognitive	9	83.3	57.6	28	54.3	11.4	4.6	66.7
Bakker, 2020	Low	Parallel	Multi-	13	Cardiovascular	26	64	58.4	35	21.6	8.9	4.3	NA
Ballester, 1999	Some	Parallel	Mono-	13	OSA symptoms	68	88.2	53	32	55	12.1	5.2	NA
Banghoj, 2020	Low	Parallel	Multi-	12	Cardiovascular	29	72	63	36.1	32	7	5.6	44
Barbe, 2012	Low	Parallel	Multi-	208	Cardiovascular	357	87.7	52	31.3	43.4	6.5	4.5	64.4
Barbe, 2010	Low	Parallel	Multi-	52	Cardiovascular	178	85	56	33	49	6.4	4.7	NA
Barbe, 2001	Low	Parallel	Multi-	6	Quality of life	29	89.7	54	29	54	7	5	NA
Barnes, 2004	Low	Cross-over	Multi-	13	OSA symptoms	80	78.8	46.4	31	21.5	10.2	3.6	43
Barnes, 2002	High	Cross-over	Multi-	8	Cardiovascular	23	85.7	46.7	31.5	13.1	11.5	3.5	47.8
Becker, 2003	Some	Parallel	Mono-	9.3	Cardiovascular	30	94	54.4	33.3	62.5	14.4	5.5	NA
Berlowitz, 2018	Some	Parallel	Multi-	13	Neurocognitive	80	91.8	46.3	NA	45.0	NA	2.9	21
Bernasconi, 2020	Low	Parallel	Multi-	52	Cardiovascular	16	78.9	64.1	28.9	36.7	NA	NA	83.3
Berry, 2011	Some	Parallel	Multi-	12	OSA symptoms	119	71.4	47.7	32.6	13.9	9.9	NA	88.2
Bigini, 2019	Some	Parallel	Mono-	12	Metabolic	4	30	61.7	35.1	20.9	8.8	5.3	NA
Borges, 2020	Low	Parallel	Mono-	8	Cardiovascular	18	61	53	31	NA	11	5.4	82
Brown, 2013	Some	Parallel	Mono-	13	Neurocognitive	15	33	61	27.3	19.4	NA	4.2	NA
Campos-Rodriguez, 2016	Low	Parallel	Multi-	13	Quality of life	151	0	58.8	33.3	36.5	10.2	4.8	75.3
Campos-Rodriguez, 2006	Low	Parallel	Mono-	4	Cardiovascular	34	55.8	55.3	35.7	58.3	15	5	NA
Caples, 2019	Some	Parallel	Mono-	52	Cardiovascular	12	58	63.5	36	30.3	4.8	6.2	NA
Carneiro-Barrera, 2022	Some	Parallel	Mono-	8	OSA symptoms	49	100	55.3	33.9	41.1	9	NA	100
Casitas, 2017	Low	Cross-over	Mono-	12	Cardiovascular	15	81	58	30	44	8.2	5.3	NA
Chasens, 2013	Low	Parallel	Mono-	4	OSA symptoms	12	58	57.6	36.2	50.2	11.4	4.9	64
Chasens, 2022	Low	Parallel	Multi-	12	Metabolic	44	53.1	60.1	35.7	22.5	10.2	4.9	NA
Chen, 2020	High	Parallel	Mono-	13	Cardiovascular	26	90	45.9	27.9	44.7	NA	4.9	69.2
Comodore, 2009	Low	Cross-over	Mono-	4	Cardiovascular	13	69.2	55.5	31.1	27.9	6.8	5.5	NA

http://doi.org/10.3390/jcm15093264

Coughlin, 2007	High	Cross-over	Mono-	6	Metabolic	34	100	49	36.1	NA	13.8	3.9	NA
Craig, 2012	Low	Parallel	Multi-	26	Cardiovascular	150	78.5	57.9	32.2	NA	7.9	2.5	NA
Craig, 2009	Low	Parallel	Mono-	4.3	Cardiovascular	43	100	49.2	36.3	NA	15.9	4.6	NA
Dalmases, 2015	Low	Parallel	Mono-	13	Neurocognitive	17	64.7	70.8	32.8	61.2	7.9	6	NA
de Oliveira, 2014	Low	Parallel	Mono-	8	Cardiovascular	24	58	59.5	30.2	21.6	11.1	5.5	NA
Diaferia, 2013	Some	Parallel	Mono-	13	Quality of life	27	100	46.4	28.7	34.4	12	3.6	NA
Drager, 2011	Low	Parallel	Mono-	13	Cardiovascular	18	100	43	28.5	55	12	5.2	NA
Durán-Cantolla, 2010	Low	Parallel	Multi-	12	Cardiovascular	169	79	53.2	31.9	44.5	10.3	4.5	65
Engleman, 1999	Some	Cross-over	Mono-	4	OSA symptoms	34	61.8	44	30	10	NA	2.8	NA
Engleman, 1998	Some	Cross-over	Mono-	4	Neurocognitive	23	91.3	47	30	43	12	2.8	NA
Eskandari, 2014	Low	Parallel	Mono-	24	OSA symptoms	11	92.3	44	31	48	14	5.2	NA
Faccenda, 2001	Some	Cross-over	Mono-	4	Cardiovascular	68	80.9	50.1	31	40.4	15	3.3	NA
Gottlieb, 2014	Low	Parallel	Mono-	12	Cardiovascular	106	68	63.5	33	25.4	8	3.5	NA
Guimaraes, 2021	Some	Parallel	Mono-	52	Cardiovascular	15	54.8	49	28.7	10	10	3.8	97
Gupta, 2018	Low	Parallel	Mono-	52	Cardiovascular	30	80	52.7	25.6	38.7	7.1	4.2	NA
Hall, 2014	Low	Parallel	Mono-	7	Cardiovascular	20	77	57.8	NA	27	10.7	4.4	NA
Hill, 2020	Low	Parallel	Multi-	4.3	OSA symptoms	14	50	29	33.2	31.8	11	3.2	35.7
Hoyos, 2024	Low	Parallel	Mono-	13	Metabolic	8	NA	NA	NA	NA	NA	5.3	NA
Hoyos, 2015	Low	Cross-over	Multi-	8	Cardiovascular	26	86.7	49.1	31.4	40	10.5	4.3	NA
Hsu, 2006	Some	Parallel	Multi-	8	Neurocognitive	15	60	76.2	25.6	43.5	8.2	1.4	NA
Huang, 2015	Some	Parallel	Mono-	156	Cardiovascular	36	77.8	62	27.9	28.3	9.3	4.5	NA
Huang, 2016	Low	Parallel	Mono-	52	Metabolic	36	80	62.4	23.1	28.5	9.4	4.2	NA
Hui, 2006	Low	Parallel	Mono-	12	Cardiovascular	28	79	50.3	27.5	32.9	10.7	5.1	NA
Hunt, 2022	Some	Parallel	Multi-	52	Cardiovascular	37	76	62	30	24	5.6	4.3	60
Ip, 2004	Low	Parallel	Mono-	4	Cardiovascular	14	100	44.4	29.6	47.7	NA	4.3	NA
Jackson, 2020	Low	Parallel	Mono-	17.3	Neurocognitive	67	56.1	52.3	35.4	NA	8	4.5	NA
Jenkinson, 1999	Low	Parallel	Mono-	4.3	OSA symptoms	52	100	50.3	35.1	NA	16.1	5	NA
Jones, 2013	Some	Cross-over	Mono-	12	Cardiovascular	43	65.1	46	29.6	30.7	11.2	3.2	39.5
Joyeux-Faure, 2018	Low	Parallel	Multi-	13	Metabolic	17	86.5	60	28.9	38.3	8.2	3.7	52.9
Joyeux-Faure, 2016	Low	Parallel	Mono-	6	Neurocognitive	15	72.2	54.9	29.6	41.5	10.4	4.5	66.7
Kaneko, 2003	Low	Parallel	Mono-	4	Cardiovascular	12	92	55.9	30.4	37.1	6.8	6.2	NA
Khadadah, 2022	Low	Parallel	Multi-	26	Neurocognitive	17	35.3	49.6	28.8	31	12.5	5.5	64.7
Kohler, 2008	Low	Parallel	Mono-	4	Cardiovascular	20	100	48.1	35.8	NA	15.8	4.7	NA
Kritikou, 2016	Low	Cross-over	Mono-	8.7	Metabolic	35	50	55.5	28.5	38.5	NA	6	NA
Krogager, 2020	Low	Parallel	Multi-	12	Cardiovascular	36	72.2	64	36.1	32	7	5.4	47.2
Kushida, 2012	Low	Parallel	Multi-	26	Neurocognitive	427	65.3	52.2	32.4	39.7	10.1	4.8	42.4
Lam, 2017	Low	Parallel	Mono-	13	Metabolic	30	81	55	29.9	43.4	7.7	2.5	40
Lam, 2007	Low	Parallel	Mono-	10	Quality of life	34	27	45	27.6	23.8	12	4.2	NA
Lao, 2022	Some	Parallel	Multi-	192	Quality of life	63	57.1	62.2	NA	24.9	7	4.4	65.1
Lloberes, 2014	Low	Parallel	Mono-	13	Cardiovascular	50	NA	NA	NA	NA	NA	5.6	75
Lojander, 1996	Some	Parallel	Mono-	52	OSA symptoms	13	95.7	50.8	30.5	NA	NA	NA	87
Lozano, 2010	Low	Parallel	Mono-	13	Cardiovascular	38	75.9	59.2	30	59.8	6.4	5.6	NA

http://doi.org/10.3390/jcm15093264

Lui, 2020	Low	Parallel	Mono-	4	Metabolic	45	77.8	46.5	28.6	61.6	10.7	NA	55.6
Lui, 2021	Low	Parallel	Mono-	8	Cardiovascular	40	78.3	52.5	30.9	49.8	8.4	3.1	56
Luz, 2023	Some	Parallel	Mono-	52	Quality of life	31	55	49	28.7	10.6	10	3.8	NA
Malow, 2008	Low	Parallel	Multi-	10	Neurocognitive	19	58	41.8	32.7	16.1	11.5	4.7	NA
Mansfield, 2004	Low	Parallel	Mono-	13	Cardiovascular	19	100	55.5	33.6	25	9.5	5.6	NA
Martinez-Ceron, 2016	Some	Parallel	Mono-	26	Metabolic	18	69	60	32.6	35.6	7.4	5.2	76.9
Martinez-Garcia, 2015	Low	Parallel	Multi-	13	Quality of life	115	63.5	75.4	33	53.5	9.6	4.9	69.6
Martínez-García, 2013	Low	Parallel	Multi-	13	Cardiovascular	98	72.4	57.8	34.3	41.3	8.9	5	72.4
May, 2018	Some	Parallel	Mono-	8	OSA symptoms	76	55	50.3	36.7	24.8	8.8	4.6	NA
McEvoy, 2016	Some	Parallel	Multi-	52	Cardiovascular	1359	81.1	61.3	28.8	29	7.3	3.5	NA
McMillan, 2014	Some	Parallel	Multi-	52	OSA symptoms	114	86	70.9	33.9	30.8	11.6	2.4	35
Melehan, 2018	Low	Parallel	Multi-	12	Cardiovascular	29	100	55.7	32.9	48.2	10.3	3.7	NA
Monasterio, 2001	Some	Parallel	Multi-	26	OSA symptoms	66	81	53	29.4	20	12.1	4.8	64
Montserrat, 2001	Some	Parallel	Mono-	6	OSA symptoms	24	91	55.7	30.3	50.5	16.1	4.3	NA
Muxfeldt, 2015	Low	Parallel	Mono-	26	Cardiovascular	62	37.9	60.8	32.9	44	10	NA	74
Nalliah, 2022	Low	Parallel	Multi-	26	Cardiovascular	12	91	59	NA	45	NA	4.5	83
Ng, 2018	Low	Parallel	Mono-	13	OSA symptoms	17	47.1	49.3	28.1	19.1	10.4	5.2	70.5
Ng, 2021	Some	Parallel	Mono-	26	Metabolic	57	51.7	55	27.5	22.5	8	4.4	NA
Ng, 2022	Some	Parallel	Mono-	26	Metabolic	66	66.7	50.8	28.9	NA	10.7	4	NA
Ng, 2016	Low	Parallel	Mono-	13	Metabolic	45	71.1	50.3	28.2	30.6	12.4	4.2	NA
Nguyen, 2010	Low	Parallel	Mono-	13	Cardiovascular	10	80	52.9	30.1	38.8	NA	5.1	NA
Parra, 2015	Low	Parallel	Multi-	103	Cardiovascular	57	72	63.7	30.2	NA	8.3	5.3	NA
Pascual, 2018	Low	Parallel	Mono-	13	Cardiovascular	30	100	NA	NA	55.8	NA	NA	56.7
Paz y Mar, 2016	Some	Parallel	Mono-	8.7	Cardiovascular	72	54.7	50.3	37.3	22.4	10	4.3	52.8
Pedrosa, 2013	Low	Parallel	Mono-	26	Cardiovascular	20	74	57	36	37.1	12	6	95
Peker, 2016	Low	Parallel	Mono-	52	Cardiovascular	76	82	65.5	28.4	28.3	5.5	5.8	NA
Pepperell, 2002	Low	Parallel	Mono-	4	Cardiovascular	53	100	50.1	34.6	NA	16.3	4.9	NA
Phillips, 2011	Low	Cross-over	Multi-	8.7	Metabolic	16	92.1	49	32.1	41.2	11.2	4.4	NA
Ponce, 2019	Low	Parallel	Multi-	13	OSA symptoms	73	68.5	74.6	31.1	22.2	9	5.2	72.6
Quan, 2013	Low	Parallel	Multi-	26	Metabolic	425	64.9	52.9	32.4	39.7	10.3	4.8	45.7
Redline, 1998	Low	Parallel	Mono-	12	Quality of life	59	59	48.1	33.4	NA	10.4	3.1	NA
Robinson, 2006	Low	Cross-over	Mono-	4.3	Cardiovascular	32	88.6	54	33.2	NA	5.1	5.2	NA
Rocha, 2023	Low	Parallel	Mono-	26	Neurocognitive	22	100	48.8	31.4	40.3	11	5.3	NA
Rosenzweig, 2016	Low	Parallel	Multi-	4.3	Neurocognitive	28	100	48.6	34.7	36.6	13.1	4.9	NA
Ruttanaumpawan, 2008	Some	Parallel	Mono-	4	Cardiovascular	19	95	59	30.3	36.2	NA	6.2	NA
Ryan, 2011	Low	Parallel	Mono-	4.3	Neurocognitive	22	72.7	62.8	28.8	38.5	4.4	5	NA
Salord, 2016	Low	Parallel	Multi-	12	Metabolic	42	26	48.5	45.7	66.3	7.9	5.4	86
Sanchez-de-la-Torre, 2020	Low	Parallel	Multi-	52	Cardiovascular	548	84	59.9	29.6	36.4	5.4	2.8	NA
Servantes, 2018	Low	Parallel	Mono-	13	Quality of life	16	69	57	31	32	9	5	NA
Shaw, 2016	Some	Parallel	Multi-	26	Metabolic	119	65.6	62.4	33.4	28	10	4.9	61.3
Shim, 2018	Low	Parallel	Mono-	13	Cardiovascular	26	92	49.1	27.8	64.2	NA	4.6	82
Silva, 2021	High	Parallel	Mono-	52	Metabolic	15	55	50.1	29.3	10	NA	3.8	NA

Simpson, 2013	Some	Parallel	Multi-	12	Cardiovascular	21	100	52.2	31.7	38.1	NA	3.5	62
Smith, 2007	Low	Cross-over	Mono-	6	OSA symptoms	23	88	61	31	36	10	3.5	NA
Spicuzza, 2006	Low	Parallel	Mono-	4	OSA symptoms	15	80	55.9	31.1	55.3	NA	6	NA
Sundar, 2020	High	Parallel	Mono-	6	OSA symptoms	9	22.2	52.4	38.3	35.4	NA	4.1	NA
Takaesu, 2012	Some	Cross-over	Mono-	4	Neurocognitive	12	91.7	41.3	26.2	40.7	NA	4.6	NA
Tang, 2020	High	Parallel	Mono-	260	Cardiovascular	43	86	58.2	32.6	NA	NA	5.1	26.6
Tantrakul, 2023	Some	Parallel	Multi-	24.4	Cardiovascular	153	0	32.9	30.1	8	8.2	2.5	32.7
Taskin, 2010	Low	Parallel	Mono-	4	Cardiovascular	17	100	52.1	NA	35	10.1	7	100
Thunström, 2017	Low	Parallel	Mono-	52	Metabolic	105	82.9	65.4	28.3	27.9	5.6	3.4	44.8
Traaen, 2021	Some	Parallel	Multi-	21.7	Cardiovascular	52	72	63	29.5	27.7	8.2	4.4	66.7
Wang, 2024	Low	Parallel	Multi-	13	Quality of life	106	46	57.1	25.3	20.6	3.6	3.3	47.5
Wang, 2020	Some	Parallel	Mono-	12	Cardiovascular	54	64.1	55.3	27.5	42.9	18.7	5.2	NA
Weaver, 2012	Low	Parallel	Mono-	8	Quality of life	121	54.5	49.5	33.2	12.8	15.2	4	NA
West, 2009	Low	Parallel	Mono-	13	Neurocognitive	16	100	57.2	37.4	NA	13.4	3.8	NA
West, 2018	Low	Parallel	Mono-	52	Neurocognitive	42	67.2	64.9	34.8	32.9	9.1	1.8	22
West, 2007	Low	Parallel	Mono-	13	Metabolic	21	100	57.8	36.6	NA	14.7	3.3	NA
Wimms, 2019	Low	Parallel	Multi-	13	Quality of life	115	70	50.6	30.3	10.6	9.9	3.8	NA
Woodson, 2003	Low	Parallel	Multi-	8	Neurocognitive	28	75	51.7	29.1	19.8	12.6	4.2	37.5
Xia, 2021	Low	Parallel	Mono-	13	Cardiovascular	26	88.5	47.2	28.2	46.9	7.1	5	53.8
Yin, 2020	Low	Parallel	Mono-	13	OSA symptoms	38	NA	41.3	28.1	59.6	9.6	NA	84
Zhao, 2022	Low	Parallel	Mono-	52	Cardiovascular	30	66.3	63.8	31.1	26.2	8	3.4	NA
Zou, 2018	Low	Parallel	Mono-	13	Cardiovascular	43	60.5	61.2	31.7	46.5	10	4.7	67.4

Table S2. Study characteristics. AHI = apnoea-hypopnea index, BMI = body mass index, CPAP = continuous positive airway pressure, ESS = Epworth Sleepiness Scale, OSA = obstructive sleep apnoea.

http://doi.org/10.3390/jcm15093264

Supplementum S1.4: Funnel plot

Figure S1. Funnel plot.

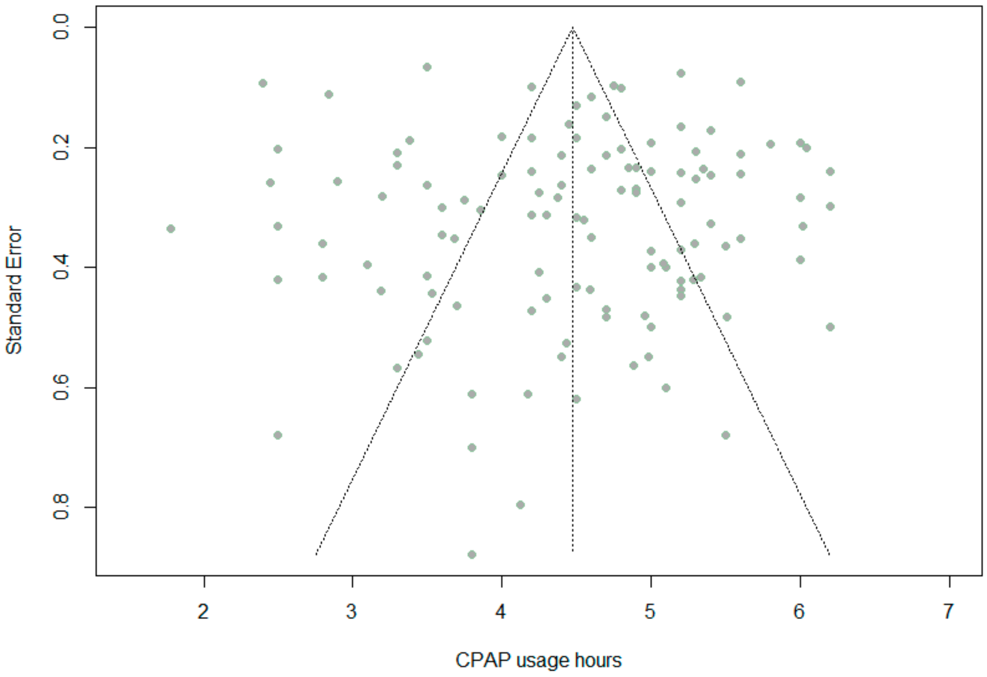

SupplementumS1.5: List of included RCTs

Aaronson, J.A., et al., *Effects of Continuous Positive Airway Pressure on Cognitive and Functional Outcome of Stroke Patients with Obstructive Sleep Apnea: A Randomized Controlled Trial.* J Clin Sleep Med, 2016. **12**(4): p. 533-41.

Alessi, C.A., et al., *Randomized controlled trial of an integrated approach to treating insomnia and improving the use of positive airway pressure therapy in veterans with comorbid insomnia disorder and obstructive sleep apnea.* Sleep, 2021. **44**(4).

Hall, A.B., et al., *Effects of short-term continuous positive airway pressure on myocardial sympathetic nerve function and energetics in patients with heart failure and obstructive sleep apnea: a randomized study.* Circulation, 2014. **130**(11): p. 892-901.

Arias, M.A., et al., *Obstructive sleep apnea syndrome affects left ventricular diastolic function: effects of nasal continuous positive airway pressure in men.* Circulation, 2005. **112**(3): p. 375-83.

Arias, M.A., et al., *Pulmonary hypertension in obstructive sleep apnoea: effects of continuous positive airway pressure: a randomized, controlled cross-over study.* Eur Heart J, 2006. **27**(9): p. 1106-13.

Aurora, R.N., et al., *Effects of Positive Airway Pressure Therapy on Glycemic Variability in Patients With Type 2 Diabetes and OSA: A Randomized Controlled Trial.* Chest, 2023. **164**(4): p. 1057-1067.

Baillieul, S., et al., *A randomized sham-controlled trial on the effect of continuous positive airway pressure treatment on gait control in severe obstructive sleep apnea patients.* Sci Rep, 2021. **11**(1): p. 9329.

Bakker, J.P., et al., *The Effect of Continuous Positive Airway Pressure on Vascular Function and Cardiac Structure in Diabetes and Sleep Apnea. A Randomized Controlled Trial.* Ann Am Thorac Soc, 2020. **17**(4): p. 474-483.

Ballester, E., et al., *Evidence of the effectiveness of continuous positive airway pressure in the treatment of sleep apnea/hypopnea syndrome.* Am J Respir Crit Care Med, 1999. **159**(2): p. 495-501.

Banghoj, A.M., et al., *Effect of 12-week continuous positive airway pressure therapy on glucose levels assessed by continuous glucose monitoring in people with type 2 diabetes and obstructive sleep apnoea; a randomized controlled trial.* Endocrinol Diabetes Metab, 2021. **4**(2): p. e00148.

Barbe, F., et al., *Effect of continuous positive airway pressure on the incidence of hypertension and cardiovascular events in nonsleepy patients with obstructive sleep apnea: a randomized controlled trial.* JAMA, 2012. **307**(20): p. 2161-8.

Barbe, F., et al., *Long-term effect of continuous positive airway pressure in hypertensive patients with sleep apnea.* Am J Respir Crit Care Med, 2010. **181**(7): p. 718-26.

Barbe, F., et al., *Treatment with continuous positive airway pressure is not effective in patients with sleep apnea but no daytime sleepiness. a randomized, controlled trial.* Ann Intern Med, 2001. **134**(11): p. 1015-23.

Barnes, M., et al., *Efficacy of positive airway pressure and oral appliance in mild to moderate obstructive sleep apnea.* Am J Respir Crit Care Med, 2004. **170**(6): p. 656-64.

Barnes, M., et al., *A randomized controlled trial of continuous positive airway pressure in mild obstructive sleep apnea.* Am J Respir Crit Care Med, 2002. **165**(6): p. 773-80.

Becker, H.F., et al., *Effect of nasal continuous positive airway pressure treatment on blood pressure in patients with obstructive sleep apnea.* Circulation, 2003. **107**(1): p. 68-73.

Berlowitz, D.J., et al., *Positive airway pressure for sleep-disordered breathing in acute quadriplegia: a randomised controlled trial.* Thorax, 2019. **74**(3): p. 282-290.

Bernasconi, C., et al., *SAS CARE 2 - a randomized study of CPAP in patients with obstructive sleep disordered breathing following ischemic stroke or transient ischemic attack.* Sleep Med X, 2020. **2**: p. 100027.

Berry, R.B., M.H. Kryger, and C.A. Massie, *A novel nasal expiratory positive airway pressure (EPAP) device for the treatment of obstructive sleep apnea: a randomized controlled trial.* Sleep, 2011. **34**(4): p. 479-85.

Bigini, E.G., et al., *DNA methylation changes and improved sleep quality in adults with obstructive sleep apnea and diabetes.* BMJ Open Diabetes Res Care, 2019. **7**(1): p. e000707.

Borges, Y.G., et al., *Oxidative stress and inflammatory profiles in obstructive sleep apnea: are short-term CPAP or aerobic exercise therapies effective?* Sleep Breath, 2020. **24**(2): p. 541-549.

Brown, D.L., et al., *Sleep apnea treatment after stroke (SATS) trial: is it feasible?* J Stroke Cerebrovasc Dis, 2013. **22**(8): p. 1216-24.

Campos-Rodriguez, F., et al., *Continuous Positive Airway Pressure Improves Quality of Life in Women with Obstructive Sleep Apnea. A Randomized Controlled Trial.* Am J Respir Crit Care Med, 2016. **194**(10): p. 1286-1294.

Campos-Rodriguez, F., et al., *Effect of continuous positive airway pressure on ambulatory BP in patients with sleep apnea and hypertension: a placebo-controlled trial.* Chest, 2006. **129**(6): p. 1459-67.

Caples, S.M., et al., *The impact of continuous positive airway pressure treatment on the recurrence of atrial fibrillation post cardioversion: A randomized controlled trial.* Int J Cardiol, 2019. **278**: p. 133-136.

Carneiro-Barrera, A., et al., *Effect of an Interdisciplinary Weight Loss and Lifestyle Intervention on Obstructive Sleep Apnea Severity: The INTERAPNEA Randomized Clinical Trial.* JAMA Netw Open, 2022. **5**(4): p. e228212.

Casitas, R., et al., *The effect of treatment for sleep apnoea on determinants of blood pressure control.* Eur Respir J, 2017. **50**(5).

Chasens, E.R., O.J. Drumheller, and P.J. Strollo, Jr., *Success in blinding to group assignment with sham-CPAP.* Biol Res Nurs, 2013. **15**(4): p. 465-9.

Chasens, E.R., et al., *Effect of Treatment of OSA With CPAP on Glycemic Control in Adults With Type 2 Diabetes: The Diabetes Sleep Treatment Trial (DSTT).* Endocr Pract, 2022. **28**(4): p. 364-371.

Chen, Q., et al., *A randomized controlled trial on ambulatory blood pressure lowering effect of CPAP in patients with obstructive sleep apnea and nocturnal hypertension.* Blood Press, 2020. **29**(1): p. 21-30.

Comondore, V.R., et al., *The impact of CPAP on cardiovascular biomarkers in minimally symptomatic patients with obstructive sleep apnea: a pilot feasibility randomized crossover trial.* Lung, 2009. **187**(1): p. 17-22.

Coughlin, S.R., et al., *Cardiovascular and metabolic effects of CPAP in obese males with OSA.* Eur Respir J, 2007. **29**(4): p. 720-7.

Craig, S.E., et al., *Continuous positive airway pressure improves sleepiness but not calculated vascular risk in patients with minimally symptomatic obstructive sleep apnoea: the MOSAIC randomised controlled trial.* Thorax, 2012. **67**(12): p. 1090-6.

Craig, S., et al., *Continuous positive airway pressure treatment for obstructive sleep apnoea reduces resting heart rate but does not affect dysrhythmias: a randomised controlled trial.* J Sleep Res, 2009. **18**(3): p. 329-36.

Dalmases, M., et al., *Effect of CPAP on Cognition, Brain Function, and Structure Among Elderly Patients With OSA: A Randomized Pilot Study.* Chest, 2015. **148**(5): p. 1214-1223.

de Oliveira, A.C., et al., *The antihypertensive effect of positive airway pressure on resistant hypertension of patients with obstructive sleep apnea: a randomized, double-blind, clinical trial.* Am J Respir Crit Care Med, 2014. **190**(3): p. 345-7.

Diaferia, G., et al., *Effect of speech therapy as adjunct treatment to continuous positive airway pressure on the quality of life of patients with obstructive sleep apnea.* Sleep Med, 2013. **14**(7): p. 628-35.

Drager, L.F., et al., *Effects of continuous positive airway pressure on early signs of atherosclerosis in obstructive sleep apnea.* Am J Respir Crit Care Med, 2007. **176**(7): p. 706-12.

Duran-Cantolla, J., et al., *Continuous positive airway pressure as treatment for systemic hypertension in people with obstructive sleep apnoea: randomised controlled trial.* BMJ, 2010. **341**: p. c5991.

Engleman, H.M., et al., *Randomized placebo-controlled crossover trial of continuous positive airway pressure for mild sleep Apnea/Hypopnea syndrome.* Am J Respir Crit Care Med, 1999. **159**(2): p. 461-7.

Engleman, H.M., et al., *Randomised placebo controlled trial of daytime function after continuous positive airway pressure (CPAP) therapy for the sleep apnoea/hypopnoea syndrome.* Thorax, 1998. **53**(5): p. 341-5.

Eskandari, D., et al., *Zonisamide reduces obstructive sleep apnoea: a randomised placebo-controlled study.* Eur Respir J, 2014. **44**(1): p. 140-9.

Faccenda, J.F., et al., *Randomized placebo-controlled trial of continuous positive airway pressure on blood pressure in the sleep apnea-hypopnea syndrome.* Am J Respir Crit Care Med, 2001. **163**(2): p. 344-8.

Gottlieb, D.J., et al., *CPAP versus oxygen in obstructive sleep apnea.* N Engl J Med, 2014. **370**(24): p. 2276-85.

Guimaraes, T.M., et al., *The treatment of mild OSA with CPAP or mandibular advancement device and the effect on blood pressure and endothelial function after one year of treatment.* J Clin Sleep Med, 2021. **17**(2): p. 149-158.

Gupta, A., et al., *Role of Positive Airway Pressure Therapy for Obstructive Sleep Apnea in Patients With Stroke: A Randomized Controlled Trial.* J Clin Sleep Med, 2018. **14**(4): p. 511-521.

Hill, E.A., et al., *Prospective Trial of CPAP in Community-Dwelling Adults with Down Syndrome and Obstructive Sleep Apnea Syndrome.* Brain Sci, 2020. **10**(11).

Hoyos, C.M., et al., *Cardio-metabolic health effects of CPAP treatment for sleep apnoea during weight loss: A randomised controlled pilot trial.* Obes Res Clin Pract, 2024. **18**(3): p. 238-241.

Hoyos, C.M., et al., *Treatment of Sleep Apnea With CPAP Lowers Central and Peripheral Blood Pressure Independent of the Time-of-Day: A Randomized Controlled Study.* Am J Hypertens, 2015. **28**(10): p. 1222-8.

Hsu, C.Y., et al., *Sleep-disordered breathing after stroke: a randomised controlled trial of continuous positive airway pressure.* J Neurol Neurosurg Psychiatry, 2006. **77**(10): p. 1143-9.

Huang, Z., et al., *Long-term effects of continuous positive airway pressure on blood pressure and prognosis in hypertensive patients with coronary heart disease and obstructive sleep apnea: a randomized controlled trial.* Am J Hypertens, 2015. **28**(3): p. 300-6.

Huang, Z., et al., *Effects of Continuous Positive Airway Pressure on Lipidaemia and High-sensitivity C-reactive Protein Levels in Non-obese Patients with Coronary Artery Disease and Obstructive Sleep Apnoea.* Heart Lung Circ, 2016. **25**(6): p. 576-83.

Hui, D.S., et al., *Nasal CPAP reduces systemic blood pressure in patients with obstructive sleep apnoea and mild sleepiness.* Thorax, 2006. **61**(12): p. 1083-90.

Hunt, T.E., et al., *Effect of continuous positive airway pressure therapy on recurrence of atrial fibrillation after pulmonary vein isolation in patients with obstructive sleep apnea: A randomized controlled trial.* Heart Rhythm, 2022. **19**(9): p. 1433-1441.

Ip, M.S., et al., *Endothelial function in obstructive sleep apnea and response to treatment.* Am J Respir Crit Care Med, 2004. **169**(3): p. 348-53.

Jackson, M.L., et al., *Does continuous positive airways pressure treatment improve clinical depression in obstructive sleep apnea? A randomized wait-list controlled study.* Depress Anxiety, 2021. **38**(5): p. 498-507.

Jenkinson, C., et al., *Comparison of therapeutic and subtherapeutic nasal continuous positive airway pressure for obstructive sleep apnoea: a randomised prospective parallel trial.* Lancet, 1999. **353**(9170): p. 2100-5.

Jones, A., et al., *The effect of continuous positive airway pressure therapy on arterial stiffness and endothelial function in obstructive sleep apnea: a randomized controlled trial in patients without cardiovascular disease.* Sleep Med, 2013. **14**(12): p. 1260-5.

Joyeux-Faure, M., et al., *Continuous Positive Airway Pressure Reduces Night-Time Blood Pressure and Heart Rate in Patients With Obstructive Sleep Apnea and Resistant Hypertension: The RHOOSAS Randomized Controlled Trial.* Front Neurol, 2018. **9**: p. 318.

Joyeux-Faure, M., et al., *Continuous positive airway pressure treatment impact on memory processes in obstructive sleep apnea patients: a randomized sham-controlled trial.* Sleep Med, 2016. **24**: p. 44-50.

Kaneko, Y., et al., *Cardiovascular effects of continuous positive airway pressure in patients with heart failure and obstructive sleep apnea.* N Engl J Med, 2003. **348**(13): p. 1233-41.

Khadadah, S., et al., *Effect of continuous positive airway pressure treatment of obstructive sleep apnea-hypopnea in multiple sclerosis: A randomized, double-blind, placebo-controlled trial (SAMS-PAP study).* Mult Scler, 2022. **28**(1): p. 82-92.

Kohler, M., et al., *CPAP and measures of cardiovascular risk in males with OSAS.* Eur Respir J, 2008. **32**(6): p. 1488-96.

Kritikou, I., et al., *Sleep apnoea and the hypothalamic-pituitary-adrenal axis in men and women: effects of continuous positive airway pressure.* Eur Respir J, 2016. **47**(2): p. 531-40.

Krogager, C., et al., *Effect of 12 weeks continuous positive airway pressure on day and night arterial stiffness and blood pressure in patients with type 2 diabetes and obstructive sleep apnea: A randomized controlled trial.* J Sleep Res, 2020. **29**(4): p. e12978.

Kushida, C.A., et al., *Effects of continuous positive airway pressure on neurocognitive function in obstructive sleep apnea patients: The Apnea Positive Pressure Long-term Efficacy Study (APPLES).* Sleep, 2012. **35**(12): p. 1593-602.

Lam, J.C.M., et al., *CPAP therapy for patients with sleep apnea and type 2 diabetes mellitus improves control of blood pressure.* Sleep Breath, 2017. **21**(2): p. 377-386.

Lam, B., et al., *Randomised study of three non-surgical treatments in mild to moderate obstructive sleep apnoea.* Thorax, 2007. **62**(4): p. 354-9.

Lao, M., et al., *The interaction among OSA, CPAP, and medications in patients with comorbid OSA and cardiovascular/cerebrovascular disease: a randomized controlled trial.* BMC Pulm Med, 2022. **22**(1): p. 99.

Lloberes, P., et al., *A randomized controlled study of CPAP effect on plasma aldosterone concentration in patients with resistant hypertension and obstructive sleep apnea.* J Hypertens, 2014. **32**(8): p. 1650-7; discussion 1657.

Lojander, J., et al., *Nasal-CPAP, surgery, and conservative management for treatment of obstructive sleep apnea syndrome. A randomized study.* Chest, 1996. **110**(1): p. 114-9.

Lozano, L., et al., *Continuous positive airway pressure treatment in sleep apnea patients with resistant hypertension: a randomized, controlled trial.* J Hypertens, 2010. **28**(10): p. 2161-8.

Lui, M.M.S., et al., *Circulating adipocyte fatty acid-binding protein is reduced by continuous positive airway pressure treatment for obstructive sleep apnea-a randomized controlled study.* Sleep Breath, 2020. **24**(3): p. 817-824.

Lui, M.M., et al., *Continuous positive airway pressure improves blood pressure and serum cardiovascular biomarkers in obstructive sleep apnoea and hypertension.* Eur Respir J, 2021. **58**(5).

Luz, G.P., et al., *Effect of CPAP vs. mandibular advancement device for excessive daytime sleepiness, fatigue, mood, sustained attention, and quality of life in patients with mild OSA.* Sleep Breath, 2023. **27**(3): p. 991-1003.

Malow, B.A., et al., *Treating obstructive sleep apnea in adults with epilepsy: a randomized pilot trial.* Neurology, 2008. **71**(8): p. 572-7.

Mansfield, D.R., et al., *Controlled trial of continuous positive airway pressure in obstructive sleep apnea and heart failure.* Am J Respir Crit Care Med, 2004. **169**(3): p. 361-6.

Martinez-Ceron, E., et al., *Effect of Continuous Positive Airway Pressure on Glycemic Control in Patients with Obstructive Sleep Apnea and Type 2 Diabetes. A Randomized Clinical Trial.* Am J Respir Crit Care Med, 2016. **194**(4): p. 476-85.

Martinez-Garcia, M.A., et al., *Obstructive sleep apnoea in the elderly: role of continuous positive airway pressure treatment.* Eur Respir J, 2015. **46**(1): p. 142-51.

Martinez-Garcia, M.A., et al., *Effect of CPAP on blood pressure in patients with obstructive sleep apnea and resistant hypertension: the HIPARCO randomized clinical trial.* JAMA, 2013. **310**(22): p. 2407-15.

May, A.M., et al., *CPAP Adherence Predictors in a Randomized Trial of Moderate-to-Severe OSA Enriched With Women and Minorities.* Chest, 2018. **154**(3): p. 567-578.

McEvoy, R.D., et al., *CPAP for Prevention of Cardiovascular Events in Obstructive Sleep Apnea.* N Engl J Med, 2016. **375**(10): p. 919-31.

McMillan, A., et al., *Continuous positive airway pressure in older people with obstructive sleep apnoea syndrome (PREDICT): a 12-month, multicentre, randomised trial.* Lancet Respir Med, 2014. **2**(10): p. 804-12.

Melehan, K.L., et al., *Randomized Trial of CPAP and Vardenafil on Erectile and Arterial Function in Men With Obstructive Sleep Apnea and Erectile Dysfunction.* J Clin Endocrinol Metab, 2018. **103**(4): p. 1601-1611.

Monasterio, C., et al., *Effectiveness of continuous positive airway pressure in mild sleep apnea-hypopnea syndrome.* Am J Respir Crit Care Med, 2001. **164**(6): p. 939-43.

Montserrat, J.M., et al., *Effectiveness of CPAP treatment in daytime function in sleep apnea syndrome: a randomized controlled study with an optimized placebo.* Am J Respir Crit Care Med, 2001. **164**(4): p. 608-13.

Muxfeldt, E.S., et al., *Effects of continuous positive airway pressure treatment on clinic and ambulatory blood pressures in patients with obstructive sleep apnea and resistant hypertension: a randomized controlled trial.* Hypertension, 2015. **65**(4): p. 736-42.

Nalliah, C.J., et al., *Impact of CPAP on the Atrial Fibrillation Substrate in Obstructive Sleep Apnea: The SLEEP-AF Study.* JACC Clin Electrophysiol, 2022. **8**(7): p. 869-877.

Ng, S.S.S., et al., *Continuous positive airway pressure for obstructive sleep apnoea does not improve asthma control.* Respirology, 2018. **23**(11): p. 1055-1062.

Ng, S.S.S., et al., *Continuous Positive Airway Pressure Does Not Improve Nonalcoholic Fatty Liver Disease in Patients with Obstructive Sleep Apnea. A Randomized Clinical Trial.* Am J Respir Crit Care Med, 2021. **203**(4): p. 493-501.

Ng, S.S.S., et al., *Effect of Weight Loss and Continuous Positive Airway Pressure on Obstructive Sleep Apnea and Metabolic Profile Stratified by Craniofacial Phenotype: A Randomized Clinical Trial.* Am J Respir Crit Care Med, 2022. **205**(6): p. 711-720.

Ng, S.S., et al., *Effects of CPAP therapy on visceral fat thickness, carotid intima-media thickness and adipokines in patients with obstructive sleep apnoea.* Respirology, 2017. **22**(4): p. 786-792.

Nguyen, P.K., et al., *Nasal continuous positive airway pressure improves myocardial perfusion reserve and endothelial-dependent vasodilation in patients with obstructive sleep apnea.* J Cardiovasc Magn Reson, 2010. **12**(1): p. 50.

Parra, O., et al., *Efficacy of continuous positive airway pressure treatment on 5-year survival in patients with ischaemic stroke and obstructive sleep apnea: a randomized controlled trial.* J Sleep Res, 2015. **24**(1): p. 47-53.

Pascual, M., et al., *Erectile dysfunction in obstructive sleep apnea patients: A randomized trial on the effects of Continuous Positive Airway Pressure (CPAP).* PLoS One, 2018. **13**(8): p. e0201930.

Paz, Y.M.H.L., et al., *Effect of Continuous Positive Airway Pressure on Cardiovascular Biomarkers: The Sleep Apnea Stress Randomized Controlled Trial.* Chest, 2016. **150**(1): p. 80-90.

Pedrosa, R.P., et al., *Effects of OSA treatment on BP in patients with resistant hypertension: a randomized trial.* Chest, 2013. **144**(5): p. 1487-1494.

Peker, Y., et al., *Effect of Positive Airway Pressure on Cardiovascular Outcomes in Coronary Artery Disease Patients with Nonsleepy Obstructive Sleep Apnea. The RICCADSA Randomized Controlled Trial.* Am J Respir Crit Care Med, 2016. **194**(5): p. 613-20.

Pepperell, J.C., et al., *Ambulatory blood pressure after therapeutic and subtherapeutic nasal continuous positive airway pressure for obstructive sleep apnoea: a randomised parallel trial.* Lancet, 2002. **359**(9302): p. 204-10.

Phillips, C.L., et al., *Continuous positive airway pressure reduces postprandial lipidemia in obstructive sleep apnea: a randomized, placebo-controlled crossover trial.* Am J Respir Crit Care Med, 2011. **184**(3): p. 355-61.

http://doi.org/10.3390/jcm15093264

Ponce, S., et al., *The role of CPAP treatment in elderly patients with moderate obstructive sleep apnoea: a multicentre randomised controlled trial.* Eur Respir J, 2019. **54**(2).

Quan, S.F., et al., *Impact of treatment with continuous positive airway pressure (CPAP) on weight in obstructive sleep apnea.* J Clin Sleep Med, 2013. **9**(10): p. 989-93.

Redline, S., et al., *Improvement of mild sleep-disordered breathing with CPAP compared with conservative therapy.* Am J Respir Crit Care Med, 1998. **157**(3 Pt 1): p. 858-65.

Robinson, G.V., et al., *Continuous positive airway pressure does not reduce blood pressure in nonsleepy hypertensive OSA patients.* Eur Respir J, 2006. **27**(6): p. 1229-35.

Rocha, B.R., et al., *What is the Effect of CPAP Treatment With Humidifier on Vocal Quality?* J Voice, 2023.

Rosenzweig, I., et al., *Changes in Neurocognitive Architecture in Patients with Obstructive Sleep Apnea Treated with Continuous Positive Airway Pressure.* EBioMedicine, 2016. **7**: p. 221-9.

Ruttanaumpawan, P., et al., *Sustained effect of continuous positive airway pressure on baroreflex sensitivity in congestive heart failure patients with obstructive sleep apnea.* J Hypertens, 2008. **26**(6): p. 1163-8.

Ryan, C.M., et al., *Influence of continuous positive airway pressure on outcomes of rehabilitation in stroke patients with obstructive sleep apnea.* Stroke, 2011. **42**(4): p. 1062-7.

Salord, N., et al., *A Randomized Controlled Trial of Continuous Positive Airway Pressure on Glucose Tolerance in Obese Patients with Obstructive Sleep Apnea.* Sleep, 2016. **39**(1): p. 35-41.

Sanchez-de-la-Torre, M., et al., *Effect of obstructive sleep apnoea and its treatment with continuous positive airway pressure on the prevalence of cardiovascular events in patients with acute coronary syndrome (ISAACC study): a randomised controlled trial.* Lancet Respir Med, 2020. **8**(4): p. 359-367.

Servantes, D.M., et al., *Effects of Exercise Training and CPAP in Patients With Heart Failure and OSA: A Preliminary Study.* Chest, 2018. **154**(4): p. 808-817.

Shaw, J.E., et al., *The Effect of Treatment of Obstructive Sleep Apnea on Glycemic Control in Type 2 Diabetes.* Am J Respir Crit Care Med, 2016. **194**(4): p. 486-92.

Shim, C.Y., et al., *Effects of continuous positive airway pressure therapy on left ventricular diastolic function: a randomised, sham-controlled clinical trial.* Eur Respir J, 2018. **51**(2).

LO, E.S., et al., *The effects of continuous positive airway pressure and mandibular advancement therapy on metabolic outcomes of patients with mild obstructive sleep apnea: a randomized controlled study.* Sleep Breath, 2021. **25**(2): p. 797-805.

Simpson, P.J., et al., *Effects of continuous positive airway pressure on endothelial function and circulating progenitor cells in obstructive sleep apnoea: a randomised sham-controlled study.* Int J Cardiol, 2013. **168**(3): p. 2042-8.

Smith, L.A., et al., *Auto-titrating continuous positive airway pressure therapy in patients with chronic heart failure and obstructive sleep apnoea: a randomized placebo-controlled trial.* Eur Heart J, 2007. **28**(10): p. 1221-7.

Spicuzza, L., et al., *Effect of treatment with nasal continuous positive airway pressure on ventilatory response to hypoxia and hypercapnia in patients with sleep apnea syndrome.* Chest, 2006. **130**(3): p. 774-9.

Sundar, K.M., et al., *A Randomized, Controlled, Pilot Study of CPAP for Patients with Chronic Cough and Obstructive Sleep Apnea.* Lung, 2020. **198**(3): p. 449-457.

Takaesu, Y., et al., *Effects of nasal continuous positive airway pressure on panic disorder comorbid with obstructive sleep apnea syndrome.* Sleep Med, 2012. **13**(2): p. 156-60.

Tang, I., et al., *Effect of CPAP on cardiovascular events in minimally symptomatic OSA: long-term follow-up of the MOSAIC randomised controlled trial.* BMJ Open Respir Res, 2020. **7**(1).

Tantrakul, V., et al., *Treatment of obstructive sleep apnea in high risk pregnancy: a multicenter randomized controlled trial.* Respir Res, 2023. **24**(1): p. 171.

Taskin, U., et al., *Erectile dysfunction in severe sleep apnea patients and response to CPAP.* Int J Impot Res, 2010. **22**(2): p. 134-9.

Thunstrom, E., et al., *CPAP Does Not Reduce Inflammatory Biomarkers in Patients With Coronary Artery Disease and Nonsleepy Obstructive Sleep Apnea: A Randomized Controlled Trial.* Sleep, 2017. **40**(11).

Traaen, G.M., et al., *Effect of Continuous Positive Airway Pressure on Arrhythmia in Atrial Fibrillation and Sleep Apnea: A Randomized Controlled Trial.* Am J Respir Crit Care Med, 2021. **204**(5): p. 573-582.

Wang, L., et al., *Community-based intervention for obstructive sleep apnea in the general population: a randomized controlled trial.* Sleep, 2024. **47**(9).

Wang, X., et al., *Continuous positive airway pressure effectively ameliorates arrhythmias in patients with obstructive sleep apnea-hypopnea via counteracting the inflammation.* Am J Otolaryngol, 2020. **41**(6): p. 102655.

Weaver, T.E., et al., *Continuous positive airway pressure treatment of sleepy patients with milder obstructive sleep apnea: results of the CPAP Apnea Trial North American Program (CATNAP) randomized clinical trial.* Am J Respir Crit Care Med, 2012. **186**(7): p. 677-83.

West, S.D., et al., *The effect of continuous positive airway pressure treatment on physical activity in patients with obstructive sleep apnoea: A randomised controlled trial.* Sleep Med, 2009. **10**(9): p. 1056-8.

West, S.D., et al., *Continuous positive airway pressure effect on visual acuity in patients with type 2 diabetes and obstructive sleep apnoea: a multicentre randomised controlled trial.* Eur Respir J, 2018. **52**(4).

West, S.D., et al., *Effect of CPAP on insulin resistance and HbA1c in men with obstructive sleep apnoea and type 2 diabetes.* Thorax, 2007. **62**(11): p. 969-74.

Wimms, A.J., et al., *Continuous positive airway pressure versus standard care for the treatment of people with mild obstructive sleep apnoea (MERGE): a multicentre, randomised controlled trial.* Lancet Respir Med, 2020. **8**(4): p. 349-358.

Woodson, B.T., et al., *A randomized trial of temperature-controlled radiofrequency, continuous positive airway pressure, and placebo for obstructive sleep apnea syndrome.* Otolaryngol Head Neck Surg, 2003. **128**(6): p. 848-61.

http://doi.org/10.3390/jcm15093264

Xia, J.H., et al., *Continuous positive airway pressure adherence and blood pressure lowering in patients with obstructive sleep apnoea syndrome and nocturnal hypertension.* Blood Press, 2021. **30**(4): p. 250-257.

Yin, G., et al., *Short-term postoperative CPAP may improve the outcomes of velopharyngeal surgery for obstructive sleep apnea.* Am J Otolaryngol, 2020. **41**(2): p. 102373.

Zhao, Y.Y., et al., *Effect of continuous positive airway pressure treatment on ambulatory blood pressures in high-risk sleep apnea patients: a randomized controlled trial.* J Clin Sleep Med, 2022. **18**(8): p. 1899-1907.

Zou, Bin et al. *"Randomized controlled trial of continuous positive airway pressure treatment of resistant hypertensive patients combined with obstructive sleep apnea / hypopnea syndrome." (2018).*
